# The interaction among obesity, Type 2 diabetes mellitus, and periodontitis in Vietnamese patients

**DOI:** 10.1002/cre2.106

**Published:** 2018-04-17

**Authors:** Thuy Anh Vu Pham, Thao Thi Phuong Tran

**Affiliations:** ^1^ Department of Periodontology, Faculty of Odonto‐Stomatology University of Medicine and Pharmacy, Ho Chi Minh City Vietnam; ^2^ Faculty of Odonto‐Stomatology University of Medicine and Pharmacy Ho Chi Minh City Vietnam

**Keywords:** obesity, periodontitis, Type 2 diabetes, Vietnamese

## Abstract

To examine the relationship between obesity, Type 2 diabetes, and periodontal disease in Vietnamese patients. The sample included 712 patients aged 18 years or older who first visited the Institute of Traditional Medicine, Ho Chi Minh City. All participants completed a questionnaire and underwent anthropometric index measurements for obesity (height, weight, waist, and hip circumferences) and had their body fat percentage measured. A full periodontal examination was performed and a fasting glycemic level was determined. Occurrence and risk of periodontal outcomes were compared across 3 different measurements of obesity (body mass index, waist–hip ratio, and body fat percentage). The prevalence of periodontitis in obese group (37.0%, 36.4%, and 24.6% by body mass index, waist–hip ratio, and body fat percentage, respectively) or Type 2 diabetic group (50.7%) was significantly higher than those without these conditions (p < .05). Subjects with obesity or Type 2 diabetes had significantly greater pocket depth and clinical attachment loss than those who are not obese or diabetic (p < .001). Multivariate logistic regression, adjusted for confounding variables, showed that the likelihood (odds ratio, OR) for periodontitis was highest in the obese and Type 2 diabetic group (OR = 4.24, CI [2.29, 7.86]; OR = 4.06, CI [2.24, 7.36]; and OR = 5.44, CI [2.94, 10.03]), followed by the obese and non‐Type 2 diabetic group (OR = 2.28, CI [1.05, 4.95]; OR = 2.02, CI [1.34, 3.56]), and then the nonobese and Type 2 diabetic group (OR = 2.20, CI [1.21, 3.98]; OR = 1.99, CI [0.93, 4.24] and OR = 5.22, CI [2.76, 9.84]) when obesity was defined by body mass index, waist–hip ratio, and body fat percentage, respectively, (p < .05). There was a significant association between obesity, Type 2 diabetes, or those with both systemic conditions and periodontitis in Vietnamese patients.

## 1 INTRODUCTION

Many systemic diseases are associated with oral conditions (Babu & Gomes, 2011; Suvan, D'Aiuto, Moles, Petrie, & Donos, 2011). Periodontitis is a common chronic oral disease characterized by a chronic inflammatory condition, and its progression is influenced by many factors, such as microorganisms, cytokines, and systemic background as well as the host's response (Marchetti et al., 2012). The prevalence of periodontitis was relatively high in American adults with 46% of having periodontitis from 2009 to 2012 (Eke et al., 2015). About half of the adults in the United Kingdom suffer from this chronic problem (Chapple, 2014), and in Vietnam, periodontal diseases affect some 97.5% of the population, according to the last national survey (Tran, Trinh, & Lam, 2002). There is an increasing concern about the possible associations between periodontitis and various systemic conditions. Among these systemic health problems, Type 2 diabetes mellitus and obesity are most relevant.

Obesity is a multifactorial disease and is becoming a worldwide epidemic with an increasing prevalence in recent years, in both adults and children. According to the Global Burden of Disease Study 2015 (GBD, 2017), 603.7 million adults and 107.7 million children were obese worldwide. In 2015, high body mass index (BMI) led to 4.0 million deaths, more than two thirds of them due to cardiovascular disease (GBD, 2017). This abnormal accumulation of fat is a leading cause of morbidity from cardiovascular disease and diabetes and becomes a socioeconomic burden in all countries (Genco, Grossi, Ho, Nishimura, & Murayama, 2005).

Type 2 diabetes is recognized as a common noncommunicable disease whose complications reduce life expectancy (Levine, 2013). Similar to obesity, diabetes prevalence has risen rapidly. In Vietnam, according a national survey in 2012, the prevalence of diabetes and prediabetes was 5.4% and 13.7%, respectively (Nguyen, 2015). In a further survey conducted on a sample of 16,282 Vietnamese subjects aged 30–69 years (5,602 men and 10,680 women), the prevalence of diabetes was 6.0% (Pham & Eggleston, 2015).

There is a triangular relation between periodontitis, obesity, and diabetes (Levine, 2013). Fat tissue is not merely a passive triglyceride reservoir of the body, but also produces a vast amount of cytokines and hormones, called adipokines or adipocytokines, especially IL‐6 and TNF‐α, appear to have the ability to modulate inflammatory activity (Eke et al., 2015). According to Maciel et al. (2016), obesity influences the subgingival microbiota composition including *Aggregatibacter actinomycetemcomitans*, *Eubacterium nodatum*, *Fusobacterium nucleatum ss vincentii*, Parvimonas micra, Prevotella intermedia, *Tannerella forsythia*, *Prevotella melaninogenica*, and *Treponema Socranskii* in periodontal health and disease. Boesing, Patino, da Silva, and Moreira (2009) found obesity to be one of the multifactorial factors associated with the occurrence of periodontitis by increased production of reactive oxygen species. Type 2 diabetes also modifies the healing activity of periodontal tissue and facilitates the development of periodontitis by modulating subgingival microbiota and altering the cellular, humoral function, and expression of genes associated with periodontal destruction (Casarin et al., 2013; Duarte et al., 2012; Ribeiro et al., 2011). Furthermore, obesity causes insulin resistance and increases the risk of diabetes. The nonesterified fatty acids secreted from adipose tissue cause insulin resistance and abnormalities in β‐cell function (Kahn, Hull, & Utzschneider, 2006). A review by Bayani, Pourali, and Keivan (2017) focusing on visfatin, an adipocytokine, hypothesized that the levels of this mediator could be a link between periodontitis and Type 2 diabetes. Although a large number of studies have evaluated the relationship of obesity or Type 2 diabetes with periodontal disease, few studies have focused on the interaction between obesity and Type 2 diabetes in periodontitis.

This is the first study that was conducted in Vietnam with the aims to investigate the interaction between these diseases. Our hypothesis is that obesity and Type 2 diabetes are risk factors for periodontitis, and both obesity and Type 2 diabetes increase the risk of periodontitis in a group of Vietnamese patients. Better understanding of this relationship may help the professionals in their efforts to prevent and treat obesity, Type 2 diabetes, and periodontal disease. Increased cooperation among medical and dental professionals is highly important in reducing the global burden of disease and maintaining patients' optimal health.

The main aims of the present study were to test the associations of diabetes and obesity with periodontitis in Vietnamese patients using three measures of obesity and six measures of periodontal conditions.

## 2 METHODS

### 2.1 Subjects

A total of 712 dentate patients aged 18 years or older who first attended the examination department at the Institute of Traditional Medicine, Ho Chi Minh City, Vietnam in 2015, were recruited into the study and underwent a periodontal examination as well as having their general health conditions (obesity and diabetes) evaluated. Thirty‐three subjects (*n* = 33) were excluded on the basis of wearing fixed orthodontic braces pregnant or lactating; having less than 14 teeth present; having Type 1 diabetes mellitus; using medicine that could influence periodontal conditions; having been treated for periodontitis in the previous 3 months; and being current smokers or past smoker in previous 5 years. The final study population consisted of 679 subjects; 182 males (26.8%) and 497 females (73.2%) between 18 and 83 years of age. This study was approved by the ethics committee of the University of Medicine and Pharmacy at Ho Chi Minh City. All subjects participated voluntarily and provided written informed consent before commencement of the study.

### 2.2 Questionnaire

Each participant was interviewed by a trained sixth‐year dental student. A structured questionnaire collected sociodemographic information such as age, gender, and educational background. Education background was recorded and classified into two categories: those who had less than or equal to 12 years of schooling and those who had post high school education. Dental habits such as daily tooth brushing (categorized as 2 times or more a day or less than 2 times a day), daily dental flossing use (categorized as yes or no), daily mouth rinse use (categorized as yes or no), and dental checkup behavior (categorized as those who regularly have dental checkups and those who never have or just visit a dentist when having problems) were also obtained.

### 2.3 Measurements of obesity

Anthropometric measurements, which included weight, height, waist and hip circumferences, and body fat percentage, were measured while subjects were wearing light clothes and no shoes, by a trained nutritional nurse from the clinic. Weight was measured in kilograms (kg) and height in centimeters (cm), and BMI was calculated in kilograms per square meter (kg/m^2^). Waist circumference was measured after the participants exhaled, using a measurement tape placed in the horizontal plane, midway between the inferior margin of the ribs and the superior border of the iliac crest. Hip circumference measurement was taken around the widest portion of the buttocks in centimeters (cm). Waist circumference was divided by hip circumference to obtain the waist–hip ratio (WHR). The body fat of the subjects was measured by the bioimpedance method, using a body fat analyzer (Omron HBF 375 Body Fat Analyzer, Omron, Japan). Obesity defined by BMI was positive at BMI ≥25 (World Health Organization, 2000). Obesity defined by WHR was positive at WHR ≥85 cm for women or WHR ≥90 cm for men (World Health Organization, 2008). Obesity defined by body fat (BF) was positive at the percentage of BF ≥25% for men and ≥35% for women (Yoon, Cho, Park, Noh, & Park, 2015).

### 2.4 Diagnosis of Type 2 diabetes

Type 2 diabetes was detected by the fasting plasma glucose measurement test. Venous blood samples were collected in the morning from subjects who fasted for at least 8 hr. People with a fasting glucose level of 126 mg/dl or above, confirmed by repeating the test on another day within a week, indicated that a person suffered from Type 2 diabetes (American Diabetes Association, 2013).

### 2.5 Periodontal examination

A full‐mouth periodontal examination was performed on all participants to assess the periodontal index, including plaque index (PI), pocket depth (PD), clinical attachment level (CAL), bleeding on probing (BOP), and gingival index (GI). PD, CAL, and BOP were recorded for all teeth in the mouth (except for the third molars) at six sites per tooth, using a UNC periodontal probe (Hu‐Friedy). PI and GI were evaluated at four sites per tooth, following the Loe (1967). All clinical data were recorded by one well‐trained examiner. Ten patients were chosen randomly to examine twice. Intraexaminer agreements in assessing PI, PD, CAL, BOP, and GI were higher than 80%. Periodontitis was categorized as mild, moderate, and severe or no periodontitis according to definition of The Centers for Disease Control and Prevention and the American Academy of Periodontology (CDC‐AAP), 2012 (Eke, Page, Wei, Thornton‐Evans, & Genco, 2012).

### 2.6 Statistical analysis

Chi‐square tests were used to examine distributional differences in age, educational background, dental habits (daily tooth brushing, daily dental flossing use, daily mouth rinse use, and dental checkup behavior), obesity (based on BMI, WHR, and BF), and Type 2 diabetes by gender and to determine the relationship between periodontitis and obesity (based on BMI, WHR, and BF) or Type 2 diabetes. Independent sample *t* tests were used to detect the mean difference in age by gender and to examine the differences of the periodontal parameters (PI, GI, BOP, PD, and CAL) between the subjects with or without obesity (based on BMI, WHR, and BF) or between the subjects with or without Type 2 diabetes. Multiple logistic regression analysis was performed using periodontitis (0: “without periodontitis” and 1: “with periodontitis”) as a dependent variable and the sociodemographic information (age, gender, and educational background), dental habits (daily tooth brushing, daily dental flossing use, daily mouth rinse use, and dental checkup behavior), and periodontal parameters (PI, GI, BOP, PD, and CAL) as independent variables. Adjusted odds ratios (OR) and corresponding 95% confidence intervals (CI) were generated for all significant variables. Statistical analysis was carried out using SPSS 19.0 (SPSS Japan, Tokyo, Japan IL) and significance was set at *p* values <.05.

## 3 RESULTS

### 3.1 Characteristics of the subjects

This study comprised a total of 679 participants including 182 males (26.8%) and 497 females (73.2%) between 18 and 83 years old with a mean age of 47.9 ± 16.7 years. The mean age in males (51.4 ± 17.5) was significantly higher than that in females (46.7 ± 16.3), (*p* = .002). The characteristics of the subjects are shown in Table 1. About 30% (31.8%) of the subjects received high school education or lower, and 68.2% of the participants had higher education levels. More than half of the subjects had regular dental visits (52.7%) and used dental floss (52.4%); 600 subjects brushed their teeth at least 2 times a day (88.4%) and 79 subjects (11.6%) brushed their teeth less than 2 times a day; 408 subjects used certain mouth rinse such as saline or commercial mouth rinse (60.1%). The number of obese subjects according to BMI, WHR, and BF were 462 (68.0%), 467 (68.8%), and 345 (50.8%), respectively. Two hundred fifteen subjects (31.7%) were diagnosed with Type 2 diabetes. There were no statistically significant differences in obesity based on BMI, WHR, BF, Type 2 diabetes, educational background, and dental habits by gender.

**Table 1:** Characteristics of the subjects

Variables	Male, *n* (%)	Female, *n* (%)	Total, *n* (%)	*p* *
Age				
≤45 years	60 (21.5%)	219 (78.5%)	279 (100%)	.011
>45 years	122 (30.5%)	278 (69.5%)	400 (100%)	
Educational background				
High school or lower	52 (24.1%)	164 (75.9%)	216 (100%)	.31
Post high school	130 (28.1%)	333 (71.9%)	463 (100%)	
Daily tooth brushing				
<2 times a day	24 (30.4%)	55 (69.6%)	79 (100%)	.50
≥2 times a day	158 (26.3%)	442 (73.7%)	600 (100%)	
Daily dental flossing use				
Yes	98 (27.5%)	258 (72.5%)	356 (100%)	.67
No	84 (26.0%)	239 (74.0%)	323 (100%)	
Daily mouth rinse use				
Yes	110 (27.0%)	298 (73.0%)	408 (100%)	.93
No	72 (26.6%)	199 (73.4%)	271 (100%)	
Dental checkup behavior				
Regular	85 (23.7%)	273 (76.3%)	358 (100%)	.07
Never or when having problems	97 (30.2%)	224 (69.8%)	321 (100%)	

* Chi‐square test; significance at *p* < .05.

### 3.2 Prevalence of periodontitis and periodontal status of subjects according to obesity

Prevalence of periodontitis and periodontal status of subjects is shown in Table 2. Two hundred nineteen patients (*n* = 219, 32.3%) had periodontitis. Obese subjects, determined by BMI, WHR, and BF, showed a prevalence of periodontitis at 37.0%, 36.4%, and 42.6%, respectively; prevalence in the nonobese group was, respectively, 22.1%, 23.1%, and 21.6%. Regardless of the definitions of obesity used, the prevalence of periodontitis in the obese group was significantly higher than that in the nonobese group (*p* < .05). The means of PD and CAL in obese subjects were significantly higher than those in nonobese subjects when obesity was defined by BMI, WHR, and BF (*p* < .001).

**Table 2:** The prevalence of periodontitis and periodontal parameters of subjects according to the three obesity measures: body mass index (BMI), waist–hip ratio (WHR), and body fat percentage (BF)

Periodontal status	BMI			WHR			BF		
	Nonobese (*n* = 217)	Obese (*n* = 462)	*p*	Nonobese (*n* = 212)	Obese (*n* = 467)	*p*	Nonobese (*n* = 334)	Obese (*n* = 345)	*p*
Periodontitis, *n* (%)	48 (22.1%)	171 (37.0%)	<.001*	49 (23.1%)	170 (36.4%)	<.001*	72 (21.6%)	147 (42.6%)	<.001*
PI, Mean ± SD	1.22 ± 0.25	1.25 ± 0.29	.15**	1.22 ± 0.26	1.25 ± 0.29	.13**	1.23 ± 0.27	1.25 ± 0.29	.56**
GI, Mean ± SD	1.076 ± 0.09	1.081 ± 0.09	.60**	1.07 ± 0.09	1.08 ± 0.10	.36**	1.08 ± 0.09	1.07 ± 0.09	.78**
BOP, Mean ± SD	6.75 ± 6.35	7.82 ± 6.78	.05**	7.18 ± 6.45	7.61 ± 6.75	.43**	6.92 ± 5.77	8.02 ± 7.38	.03**
PD (mm), Mean ± SD	2.32 ± 0.82	2.61 ± 0.53	<.001**	2.39 ± 0.82	2.58 ± 0.55	.001**	2.36 ± 0.64	2.67 ± 0.63	<.001**
CAL (mm), Mean ± SD	2.34 ± 0.78	2.60 ± 0.54	<.001**	2.41 ± 0.78	2.57 ± 0.55	.002**	2.41 ± 0.64	2.63 ± 0.61	<.001**

*Note*. PI = plaque index; GI = gingival index; BOP = bleeding on probing; PD = pocket depth; CAL = clinical attachment loss.

* Chi‐square test.

** Independent *t* test, *p* < .05; significance at *p* < .05.

### 3.3 Prevalence of periodontitis and periodontal status of subjects according to Type 2 diabetes status

Prevalence of periodontitis and periodontal status of subjects according to Type 2 diabetes status is shown in Table 3. Subjects with Type 2 diabetes had a prevalence of periodontitis of 50.7%, which was significantly higher compared with 23.7% (*p* < .001) in non‐Type 2 diabetic subjects. Means of BOP, PD, and CAL in Type 2 diabeticsubjects were 9.84, 2.82, and 2.80, respectively, which were significantly higher than those in non‐Type 2 diabetic subjects (6.38, 2.38, and 2.39, respectively, [*p* < .001]).

**Table 3:** The prevalence of periodontitis and periodontal parameters of subjects according to Type 2 diabetic status

Periodontal status	Non‐Type 2 diabetic (*n* = 464)	Type 2 diabetic (*n* = 215)	*p*
Periodontitis, *n* (%)	110 (23.7%)	109 (50.7%)	<.001*
PI, Mean ± SD	1.25 ± 0.29	1.22 ± 0.26	.15**
GI, Mean ± SD	1.08 ± 0.09	1.08 ± 0.10	.31**
BOP, Mean ± SD	6.38 ± 5.92	9.84 ± 7.52	<.001**
PD (mm), Mean ± SD	2.38 ± 0.61	2.82 ± 0.63	<.001**
CAL (mm), Mean ± SD	2.39 ± 0.61	2.80 ± 0.59	<.001**

*Note*. PI = plaque index; GI = gingival index; BOP = bleeding on probing; PD = pocket depth; CAL = clinical attachment loss.

* Chi‐square test.

** Independent *t* test; significance at *p* < .05.

### 3.4 The association between periodontitis and obesity and Type 2 diabetes

The multivariate logistic regression model presented in Table 4 was carried out to assess the association between the occurrence of periodontitis, Type 2 diabetes, and obesity with the adjustment for gender, educational background; daily tooth brushing; dental flossing use; daily mouth rinse use; dental check‐up behavior; mouth rinse use; PI, GI, and PD. To determine whether Type 2 diabetes was an interacting variable in the multivariate logistic regression model, we considered the relationship between obesity and Type 2 diabetes. Obesity interacts with Type 2 diabetes when obesity definition was based on BMI, WHR, and BF. The OR for periodontitis was highest in the obese and Type 2 diabetic group (OR = 4.24, CI [2.29, 7.86]; OR = 4.06, CI [2.24, 7.36] and OR = 5.44, CI [2.94, 10.03]), followed by the obese and non‐Type 2 diabetic group (OR = 2.28, CI [1.05, 4.95]; OR = 2.02, CI [1.34, 3.56] and OR = 5.33, CI [3.05, 9.35]) and then, the nonobese and Type 2 diabetic group (OR = 2.20, CI [1.21, 3.98]; OR = 1.99, CI [0.93, 4.24] and OR = 5.22, CI [2.76, 9.84]) when obesity was defined by BMI, WHR, and BF, respectively (*p* < .05).

**Table 4:** Logistic regression for periodontitis according to the three obesity measures: body mass index (BMI), waist–hip ratio (WHR), and body fat percentage (BF)

Variables	OR (95% CI)	*p* *	Variables	OR (95% CI)	*p* *	Variables	OR (95% CI)	*p* *
Age			Age			Age		
≤45 years	1	<.001	≤45 years	1	.001	≤45 years	1	<.001
>45 years	2.14 (1.40–3.27)		>45 years	2.10 (1.37–3.22)		>45 years	2.31 (1.49–3.58)	
BOP			BOP			BOP		
Mean ≤ 7.6	1	<.001	Mean ≤ 7.6	1	<.001	Mean ≤ 7.6	1	<.001
Mean > 7.6	2.29 (1.56–3.37)		Mean > 7.6	2.37 (1.62–3.49)		Mean > 7.6	2.58 (1.74–3.84)	
CAL			CAL			CAL		
Mean ≤ 2.5	1	.035	Mean ≤ 2.5	1	.028	Mean ≤ 2.5	1	.034
Mean > 2.5	2.64 (1.08–6.46)		Mean > 2.5	2.74 (1.11–6.67)		Mean > 2.5	2.62 (1.08–6.37)	
BMI			WHR			BF		
BMI (−) Diabetes (−)	1		WHR (−) Diabetes (−)	1		BF (−) Diabetes (−)	1	
BMI (−) Diabetes (+)	2.20 (1.21–3.98)	.037	WHR (−) Diabetes (+)	1.99 (0.93–4.24)	.076	BF (−) Diabetes (+)	5.22 (2.76–9.84)	<.001
BMI (+) Diabetes (−)	2.28(1.05–4.95)	.001	WHR (+) Diabetes (−)	2.02 (1.14–3.56)	.015	BF (+) Diabetes (−)	5.33 (3.05–9.35)	<.001
BMI (+) Diabetes (+)	4.24 (2.29–7.86)	<.001	WHR (+) Diabetes (+)	4.06 (2.24–7.36)	<.001	BF (+) Diabetes (+)	5.44 (2.94–10.03)	<.001

*Note*. Logistic regression analysis. Adjusted by gender, educational background; daily tooth brushing; dental flossing use; daily mouth rinse use; dental checkup behavior; plaque index; gingival index and pocket depth. OR = odds ratio; CI = confidence interval; BOP = bleeding on probing; CAL = clinical attachment loss; (−) or (+) = “with” or “without” in each obesity status or Type 2 diabetes.

* significance at *p* < .05.

## 4 DISCUSSION

To the authors' knowledge, this is the first study to show a positive association between obesity, Type 2 diabetes, and periodontitis in Vietnamese patients. The full mouth was examined for assessment of periodontitis. Pocket depth and CAL were measured at six sites per tooth, using UNC periodontal probe with a precision of 1 mm in this study. The presence of BOP and PD implies the existence of current inflammation in periodontal tissue. In contrast, the presence of CAL suggests an accumulation of periodontal tissue breakdown attributable to periodontitis but does not necessarily correspond to current periodontal inflammation. In the present study, both of these models diagnosed periodontitis from the evaluation of BOP, PD, and CAL.

Currently, BMI is still the most popular indicator for obesity diagnosis because of its simple, practical, and noninvasive method. BMI is widely used in many studies due to the association with the risk of morbidity and mortality in obese people. However, BMI does not accurately assess the condition of excess body fat as well as lean mass or fat mass, two main components of weight that may differ between individuals (Yoon et al., 2015). In this study, in addition to using BMI, we assessed obesity by other anthropometric indicators such as the WHR. WHR has been shown to be good indicator of abdominal adiposity and helps predict disease risk better than BMI (Yusuf et al., 2005). We also used the Omron HBF‐375 Karada Scan Body Fat Analyzer (BF) that is a body‐composition‐measuring device. Body fat percentage measured through body composition is theoretically better for evaluating excess adiposity than BMI. However, many techniques for measuring body composition in vivo and are not feasible or inaccurate in epidemiological research settings (World Health Organization, 2004).

Our study showed that periodontitis prevalence in the obese group of patients, based on BMI, WHR, and BF were, respectively, 37.0%, 36.4%, and 42.6%. These levels were almost equivalent to the study performed by Lee, Yi, and Bae (2013) on 18,210 Korean adults. In our study, the periodontal status in the obese subjects was poorer than that in the nonobese group. This result is similar to other studies, which showed that the PD and CAL index in the obese groups were significantly higher than those in the nonobese groups (Buduneli et al., 2014; Khader, Bawadi, Haroun, Alomari, & Tayyem, 2009; Sarlati, Akhondi, Ettehad, Neyestani, & Kamali, 2008). The higher frequency of periodontitis, PD, and CAL index in the obesity group suggests that the severity of periodontal disease could be associated with obesity in this study.

Obese subjects, determined by BMI, WHR, and BF had higher odd ratios for the occurrence of periodontitis than did the nonobese. These results were similar to the findings of many reports on obesity and periodontal disease, suggesting that obesity has an association with periodontitis (Han, Lim, Sun, Paek, & Kim, 2010; Khader et al., 2009; Pataro et al., 2012; Pham & Nguyen, 2015). Through the fifth Korea National Health and Nutrition Examination Survey, Kim and Kim (2016) confirmed that the risk of periodontitis was increased with BMI measurement. Our findings supported the hypothesis of the existence of this relationship. However, Torrungruang et al. (2005) reported that there was no association between obesity and periodontitis. The differences between studies may come from the selection of samples. All the participants of the Thailand study had periodontitis, which was categorized as mild, moderate, and severe. This could result in an underevaluated likelihood of associated risk (OR). In a further study, De Castilhos et al. (2012) suggested a statistically significant association between obesity and gingivitis in regard to calculus and gingival bleeding. However, periodontitis was not significantly associated with obesity. Similarly, Kawabata et al. (2016) did not find an association between periodontitis and obesity—based on BMI, although this was a prospective cohort study, it was conducted on 2,588 Japanese students.

The prevalence of diabetes in our study was 31.7% of all patients examined. It was a higher prevalence than community‐based previous research in Vietnam (Nguyen, 2015; Pham & Eggleston, 2015). In other studies, diabetes was reported as a 3.7% prevalence reported by Al‐Zahrani, Bissada, and Borawski (2003), and 17.9%, by Khader et al. (2009). Besides the sample size and race difference, the higher prevalence of diabetes in our study may result from the study sample being drawn from a hospital that examined and treated many diabetic patients.

Our research showed that the periodontitis prevalence and the means of BOP, PD, and CAL in a diabetic group of patients were significantly higher than those in a nondiabetic group of patients attending the same hospital. The findings from this study was similar to those of Mealey et al. (2006), who reported that the prevalence of periodontitis in Type 2 diabetes patients was significantly higher than that in the normal population and that Type 2 diabetes was positively correlated with periodontal destruction. Increased advanced glycation end products in diabetes is involved in accelerating inflammation response and changing the tissue repair process, which affects the elasticity of gingival collagen and blood vessels (Levine, 2013; Marchetti et al., 2012). In addition, periodontal pathogens in the oral microbial flora of diabetic subjects, which is accumulating due to a higher concentration of glucose in saliva and crevicular fluid, also contribute to the severity of periodontal status. Campus, Salem, Uzzau, Baldoni, and Tonolo (2005) showed that levels of *Porphyromonas gingivalis* and *Tannerella forsythensis*, two species that cause periodontal disease in the diabetic group, were 1.2 times higher than that in nondiabetic subjects. In a recent study by Zuk, Quiñonez, Lebenbaum, and Rosella (2017), the authors concluded that glucose disruption measured by fasting plasma glucose was associated with periodontitis among Canadian adults. With the more aggravated periodontal status and higher periodontitis prevalence in diabetic subjects, our study confirmed again the association between diabetes and periodontal diseases.

In our study, we found an interaction between obesity and Type 2 diabetes that impacted on the relationship with periodontitis. We found that subjects who had only obesity or only Type 2 diabetes had a higher risk of periodontitis than those who had neither. The subjects who were obese and had Type 2 diabetes not only had a higher risk for periodontitis than the ones who had neither disease but also had more chance to have periodontitis than those who had only obesity or only Type 2 diabetes. Both obesity and Type 2 diabetes affect periodontal health through immune responses from proinflammatory cytokines (Levine, 2013). The synergistic effects of these two conditions may lead to higher risk of periodontal inflammation in subjects who have both diseases. However, in addition to systemic factors, periodontitis occurs directly as an effect of microorganisms. Thus, in patients who have both obesity and diabetes, good oral hygiene is necessary to control dental plaque and decrease the risk of periodontal disease. Furthermore, systemic treatment of these diseases could contribute to improving periodontal status. Moritz and Mealy (2006) suggested that the risk of periodontitis decreased with good glycemic control. They highlighted the need for clinicians to adjust glycemic assessment in the examination routine when periodontitis was diagnosed. In our study, the subjects who had only obesity had a higher risk of periodontitis than those who had only Type 2 diabetes. This study suggests that the association of periodontitis and obesity was stronger than that with Type 2 diabetes. We also found that obesity associated with BF had the highest risk for periodontitis. BF percentage may be the most significant indicator in the link between obesity and periodontitis in this study. Han et al. (2010) concluded that the visceral fat area was the most appropriate indicator of obesity in relation to periodontitis and that obesity could act as a substantial risk factor for periodontitis. Findings from the study of Salekzamani, Shirmohammadi, Rahbar, Shakouri, and Nayebi (2011) on 150 males showed a statistically significant association between periodontitis and BMI, WC, and body composition but not for history of diabetes. Lula, Ribeiro, Hugo, Alves, and Silva (2014) also showed that periodontitis was associated with high BMI and self‐reported diabetes, but the latter association did not remain significant in adjusted models. Levine (2013) stated that there was a triangular, self‐generating cycle of morbidity linking obesity, diabetes, and periodontal disease. Both diseases, obesity and diabetes mellitus, contributed to the progression and occurrence of periodontitis. Therefore, it is extremely challenging to clarify the causal relationship among these diseases.

Periodontal conditions may significantly be associated with the level of education, frequency of tooth brushing, regular dental checkups, and mouth rinse use (Pham et al., 2011; Pham, Kieu, & Ngo, 2017). However, we did not find any relationship between periodontitis and these variables in this study.

Although this study gives some insight into the interaction among obesity, Type 2 diabetes, and periodontitis, it has limitations. The study is cross‐sectional, and the sample was collected in a major hospital; therefore, it will not represent the general population, and cause–effect relationships cannot be inferred. In addition, some systemic diseases were not controlled as potential confounders, such as hypertension, rheumatism, or osteoporosis, which may have affected the periodontal status. A longitudinal study with a larger sample‐size based on the general population is also needed to clarify the direction and strengths of the triangular relationships between obesity, Type 2 diabetes, and periodontitis. The use of multiple estimates of obesity is a further important element to include in future studies.

#### 4.1.1. Scientific rationale for study

The association among obesity, Type 2 diabetes, and periodontitis have been reported in many studies. The triangular relationship of these three conditions was also supported in some scientific articles; however, more research is needed to clarify these associations, especially in the Asian population. To date, there is little data related to obesity, Type 2 diabetes, and periodontitis in Vietnamese populations.

#### 4.1.2. Principal findings

This study found that there is an association between obesity, Type 2 diabetes, and periodontitis in Vietnamese patients attending a Traditional Medicine Institute. The interaction of these three conditions has also been reported elsewhere.

#### 4.1.3. Practical implications

This study highlights, for dental practitioners, the clinical evidence of the relationships between obesity, diabetes, and periodontal disease and the need for an anthropometric measurement and glycemic tests in clinical examinations of patients with periodontal disease. The prevention and treatment of periodontal diseases that dental practitioners provide for patients may help reduce the adverse effect of these diseases. Furthermore, treatment of obesity and Type 2 diabetes could contribute to improving periodontal status and periodontal health.

## CONFLICT OF INTEREST

There are no conflict of interests to declare.

## References

[cre2106-bib-0001] Al‐Zahrani, M. S. , Bissada, N. F. , & Borawski, E. A. (2003). Obesity and periodontal disease in Young, middle‐aged, and older adults. Journal of Periodontology, 74, 610–615. 12816292 10.1902/jop.2003.74.5.610

[cre2106-bib-0002] American Diabetes Association (2013). Diagnosis and classification of diabetes mellitus. Diabetes Care, 36, dc13–S067.

[cre2106-bib-0003] Babu, N. C. , & Gomes, A. J. (2011). Systemic manifestations of oral diseases. Journal of Oral and Maxillofacial Pathology, 15, 144–147. 22529571 10.4103/0973-029X.84477 PMC3329699

[cre2106-bib-0046] Bayani, M. , Pourali, M. , & Keivan, M. (2017). Possible interaction between visfatin, periodontal infection, and other systemic diseases: A brief review of literature. European Journal of Dentistry, 11, 407–410. 28932156 10.4103/ejd.ejd_284_16 PMC5594975

[cre2106-bib-0004] Boesing, F. , Patino, J. S. , da Silva, V. R. G. , & Moreira, E. A. (2009). The interface between obesity and periodontitis with emphasis on oxidative stress and inflammatory response. Obesity Reviews, 10, 290–297. 19207875 10.1111/j.1467-789X.2008.00555.x

[cre2106-bib-0005] Buduneli, N. , Bıyıkoğlu, B. , İlgenli, T. , Buduneli, E. , Nalbantsoy, A. , Saraç, F. , & Kinane, D. F. (2014). Is obesity a possible modifier of periodontal disease as a chronic inflammatory process? A case–control study. Journal of Periodontal Research, 49, 465–471. 23919737 10.1111/jre.12125

[cre2106-bib-0006] Campus, G. , Salem, A. , Uzzau, S. , Baldoni, E. , & Tonolo, G. (2005). Diabetes and periodontal disease: A case‐control study. Journal of Periodontology, 76, 418–425. 15857077 10.1902/jop.2005.76.3.418

[cre2106-bib-0007] Casarin, R. C. , Barbagallo, A. , Meulman, T. , Santos, V. R. , Sallum, E. A. , Nociti, F. H. , … Gonçalves, R. B. (2013). Subgingival biodiversity in subjects with uncontrolled type‐2 diabetes and chronic periodontitis. Journal of Periodontal Research, 48, 30–36. 22762355 10.1111/j.1600-0765.2012.01498.x

[cre2106-bib-0008] Castilhos, E. D. , Horta, B. L. , Gigante, D. P. , Demarco, F. F. , Peres, K. G. , & Peres, M. A. (2012). Association between obesity and periodontal disease in young adults: A population‐based birth cohort. Journal of Clinical Periodontology, 39, 717–724. 22671969 10.1111/j.1600-051X.2012.01906.x PMC3468720

[cre2106-bib-0009] Chapple, I. L. C. (2014). Time to take periodontitis seriously. BMJ, 348, 2645. 10.1136/bmj.g2645 24721751

[cre2106-bib-0010] Duarte, P. M. , Miranda, T. S. , Lima, J. A. , Dias Gonçalves, T. E. , Santos, V. R. , Bastos, M. F. , & Ribeiro, F. V. (2012). Expression of immune‐inflammatory markers in sites of chronic periodontitis in patients with type 2 diabetes. Journal of Periodontology, 83, 426–434. 21859322 10.1902/jop.2011.110324

[cre2106-bib-0011] Eke, P. I. , Dye, B. A. , Wei, L. , Slade, G. D. , Thornton‐Evans, G. O. , Borgnakke, W. S. , … Genco, R. J. (2015). Update on prevalence of periodontitis in adults in the United States: NHANES 2009 to 2012. Journal of Periodontology, 86, 611–622. 25688694 10.1902/jop.2015.140520 PMC4460825

[cre2106-bib-0012] Eke, P. I. , Page, R. C. , Wei, L. , Thornton‐Evans, G. , & Genco, R. J. (2012). Update of the case definitions for population‐based surveillance of periodontitis. Journal of Periodontology, 83, 449–1454. 10.1902/jop.2012.110664 PMC6005373 22420873

[cre2106-bib-0013] Genco, R. J. , Grossi, S. G. , Ho, A. , Nishimura, F. , & Murayama, Y. (2005). A proposed model linking inflammation to obesity, diabetes, and periodontal infections. Journal of Periodontology, 76, 2075–2084. 10.1902/jop.2005.76.11-S.2075 16277579

[cre2106-bib-0014] Global Burden of Disease (GBD 2015) Obesity Collaborators (2017). Health effects of overweight and obesity in 195 countries over 25 years. The New England Journal of Medicine, 377, 13–27. 28604169 10.1056/NEJMoa1614362 PMC5477817

[cre2106-bib-0015] Han, D. H. , Lim, S. Y. , Sun, B. C. , Paek, D. M. , & Kim, H. D. (2010). Visceral fat area‐defined obesity and periodontitis among Koreans. Journal of Clinical Periodontology, 37, 172–179. 20041978 10.1111/j.1600-051X.2009.01515.x

[cre2106-bib-0016] Kawabata, Y. , Ekuni, D. , Miyai, H. , Kataoka, K. , Yamane, M. , Mizutani, S. , … Morita, M. (2016). Relationship betweenprehypertension/hypertension and periodontal disease: A prospectivecohort study. American Journal of Hypertension, 29, 388–396. 26208668 10.1093/ajh/hpv117

[cre2106-bib-0045] Kahn, S. E. , Hull, R. L. , & Utzschneider, K. M. (2006). Mechanisms linking obesity to insulin resistance and type 2 diabetes. Nature, 444, 840–846. 17167471 10.1038/nature05482

[cre2106-bib-0017] Khader, Y. S. , Bawadi, H. A. , Haroun, T. F. , Alomari, M. , & Tayyem, R. F. (2009). The association between periodontal disease and obesity among adults in Jordan. Journal of Clinical Periodontology, 36, 18–24. 19046327 10.1111/j.1600-051X.2008.01345.x

[cre2106-bib-0018] Kim, Y. S. , & Kim, J. H. (2016). Body mass index and oral health status in Korean adults: The fifth Korea national health and nutrition examination survey. International Journal Dental Hygiene, 15, 172–178. https://doi.org/10.1111/idh.12207 10.1111/idh.12207 26842672

[cre2106-bib-0019] Lee, J. B. , Yi, H. Y. , & Bae, K. H. (2013). The association between periodontitis and dyslipidemia based on the fourth Korea national health and nutrition examination survey. Journal of Clinical Periodontology, 40, 437–442. 23480442 10.1111/jcpe.12095

[cre2106-bib-0020] Levine, R. S. (2013). Obesity, diabetes and periodontitis, a triangular relationship? British Dental Journal, 215, 35–39. 23846063 10.1038/sj.bdj.2013.627

[cre2106-bib-0021] Loe, H. (1967). The gingival index, the plaque index and the retention index systems. Journal of Periodontology, 38(6), 610–616. 10.1902/jop.1967.38.6.610 5237684

[cre2106-bib-0022] Lula, E. C. , Ribeiro, C. C. , Hugo, F. N. , Alves, C. M. , & Silva, A. A. (2014). Added sugars and periodontal disease in young adults: An analysis of NHANES III data. The American Journal of Clinical Nutrition, 100, 1182–1187. 25240081 10.3945/ajcn.114.089656

[cre2106-bib-0023] Maciel, S. S. , Feres, M. , Gonçalves, T. E. , Zimmermann, G. S. , da Silva, H. D. , Figueiredo, L. C. , & Duarte, P. M. (2016). Does obesity influence the subgingival microbiota composition in periodontal health and disease? Journal of Clinical Periodontology, 43, 1003–1012. 27717180 10.1111/jcpe.12634

[cre2106-bib-0024] Marchetti, E. , Monaco, A. , Procaccini, L. , Mummolo, S. , Gatto, R. , Tetè, S. , … Marzo, G. (2012). Periodontal disease: The influence of metabolic syndrome. Nutrition & Metabolism, 9, 88. 23009606 10.1186/1743-7075-9-88 PMC3499456

[cre2106-bib-0025] Mealey, B. L. , Oates, T. W. , & American Academy of Periodontology (2006). Diabetes mellitus and periodontal diseases. Journal of Periodontology, 77, 1289–1303. 16881798 10.1902/jop.2006.050459

[cre2106-bib-0026] Moritz, A. , & Mealy, B. (2006). Periodontal disease, insulin resistance, and diabetes mellitus: A review and clinical implications. Grand Rounds in Oral‐Systemic Medicine, 2, 13–20.

[cre2106-bib-0027] Nguyen, T. K. (2015). Diabetes in Vietnam. Annals of Global Health, 81(6), 870–873. 27108154 10.1016/j.aogh.2016.01.003

[cre2106-bib-0028] Pataro, A. L. , Costa, F. O. , Cortelli, S. C. , Cortelli, J. R. , Abreu, M. H. , & Costa, J. E. (2012). Association between severity of body mass index and periodontal condition in women. Clinical Oral Investigation, 16, 727–734. 10.1007/s00784-011-0554-7 21556849

[cre2106-bib-0029] Pham, N. M. , & Eggleston, K. (2015). Diabetes prevalence and risk factors among Vietnamese adults: Findings from community‐based screening programs. Diabetes Care, 38, e77–e78. 25908162 10.2337/dc14-3093

[cre2106-bib-0030] Pham, T. A. V. , Kieu, T. Q. , Ngo, L. T. Q. (2017). Risk factors of periodontal disease in Vietnamese patients. Journal of Investigative and Clinical Dentistry. https://doi.org/10.1111/jicd.12272. Epub 2017 May 12. 10.1111/jicd.12272 28497901

[cre2106-bib-0031] Pham, T. A. V. , & Nguyen, N. T. X. (2015). Relationship between obesity and periodontal status in Vietnamese patients: A pilot study. International Journal of Experimental Dental Science, 4, 119–123.

[cre2106-bib-0032] Pham, T. A. V. , Ueno, M. , Shinada, K. , Yanagisawa, T. , Wright, F. A. , & Kawaguchi, Y. (2011). Periodontal disease and related factors among Vietnamese dental patients. Oral Health and Preventive Dentistry, 9, 185–194. 21842021

[cre2106-bib-0033] Ribeiro, F. V. , de Mendoncxa, A. C. , Santos, V. R. , Bastos, M. F. , Figueiredo, L. C. , & Duarte, P. M. (2011). Cytokines and bone‐related factors in systemically healthy patients with chronic periodontitis and patients with type 2 diabetes and chronic periodontitis. Journal of Periodontology, 82, 1187–1196. 21284550 10.1902/jop.2011.100643

[cre2106-bib-0034] Salekzamani, Y. , Shirmohammadi, A. , Rahbar, M. , Shakouri, S. , & Nayebi, F. (2011). Association between human body composition and periodontal disease. International Scholarly Research Network Dentistry. 863847 10.5402/2011/863847 PMC3216386 22111011

[cre2106-bib-0035] Sarlati, F. , Akhondi, N. , Ettehad, T. , Neyestani, T. , & Kamali, Z. (2008). Relationship between obesity and periodontal status in a sample of young Iranian adults. International Dental Journal, 58(1), 36–40. 18350852

[cre2106-bib-0036] Suvan, J. , D'Aiuto, F. , Moles, D. R. , Petrie, A. , & Donos, N. (2011). Association between over‐ weight/obesity and periodontitis in adults. A systematic review. Obesity Reviews, 12, 381–404. 10.1111/j.1467-789X.2010.00808.x 21348914

[cre2106-bib-0037] Torrungruang, K. , Tamsailom, S. , Rojanasomsith, K. , Sutdhibhisal, S. , Nisapakultorn, K. , Vanichjakvong, O. , … Sritara, P. (2005). Risk indicators of periodontal disease in older Thai adults. Journal of Periodontology, 76, 558–565. 15857096 10.1902/jop.2005.76.4.558

[cre2106-bib-0038] Tran, V. T. , Trinh, D. H. , & Lam, N. A. (2002). National oral health survey of Vietnam (p. 80). Hanoi, Vietnam: Medical publishing house.

[cre2106-bib-0039] World Health Organization (2000). The Asia pacific perspective: Redefining. Obesity, 16–26.

[cre2106-bib-0040] World Health Organization (2004). Appropriate body‐mass index for Asian populations and its implications for policy and intervention strategies. Lancet, 363, 157–163. 14726171 10.1016/S0140-6736(03)15268-3

[cre2106-bib-0041] World Health Organization (2008). Waist circumference and waist–hip ratio: Report of a WHO expert consultation. Geneva. 8–11.

[cre2106-bib-0042] Yoon, J. L. , Cho, J. J. , Park, K. M. , Noh, H. M. , & Park, Y. S. (2015). Diagnostic performance of body mass index using the western pacific regional office of world health organization reference standards for body fat percentage. Journal of Korean Medical Science, 30, 162–166. 25653487 10.3346/jkms.2015.30.2.162 PMC4310942

[cre2106-bib-0043] Yusuf, S. , Hawken, S. , Ounpuu, S. , Bautista, L. , Franzosi, M. G. , Commerford, P. , … INTERHEART Study Investigators (2005). Obesity and the risk of myocardial infarction in 27,000 participants from 52 countries: A case‐control study. Lancet, 366, 1640–1649. 16271645 10.1016/S0140-6736(05)67663-5

[cre2106-bib-0044] Zuk, A. , Quiñonez, C. , Lebenbaum, M. , & Rosella, L. C. (2017). The association between undiagnosed glycaemic abnormalities and cardiometabolic risk factors with periodontitis: Results from 2007–2009 Canadian Health Measures Survey. Journal of Clinical Periodontology, 44, 132–141. 28028834 10.1111/jcpe.12684

